# Experimental Antioxidant Therapy in Ataxia Telangiectasia

**DOI:** 10.4137/cmo.s535

**Published:** 2008-05-20

**Authors:** Ramune Reliene, Robert H. Schiestl

**Affiliations:** 1 Department of Pathology and Laboratory Medicine, David Geffen School of Medicine, University of California Los Angeles, Los Angeles, CA 90095, U.S.A; 2 Department of Medicine, Center for Human Nutrition, David Geffen School of Medicine, University of California Los Angeles, Los Angeles, CA 90095, U.S.A; 3 Department of Radiation Oncology, David Geffen School of Medicine, University of California Los Angeles, Los Angeles, CA 90095, U.S.A; 4 Department of Environmental Health, School of Public Health, University of California Los Angeles, Los Angeles, CA 90095, U.S.A

**Keywords:** ataxia telangiectasia, *Atm*, mouse, antioxidants, chemoprevention, lymphoma

## Abstract

Ataxia telangiectasia (AT) is a rare genetic disorder characterized by immunodeficiency, early onset neurological degeneration, hypersensitivity to ionizing radiation and a high incidence of lymphoid cancers. The disease results from bi-allelic mutations in the *AT mutated* (*ATM*) gene involved in cell cycle checkpoint control and repair of DNA double-strand breaks. Evidence has been accumulating that oxidative stress is associated with AT and may be involved in the pathogenesis of the disease. This led to a hypothesis that antioxidant therapy may mitigate the symptoms of AT, especially neurological degeneration and tumorigenesis. Consequently, several studies examined the effect of antioxidants in *Atm* deficient mice used as an animal model of AT. N-acetyl-L-cysteine (NAC), EUK-189, tempol and 5-carboxy-1,1,3,3-tetramethylisoindolin-2-yloxyl (CTMIO) have been tested for their chemopreventive properties and had some beneficial effects. In addition to antioxidants, cancer therapeutic agent dexamethasone was examined for cancer prevention in *Atm* deficient mice. Of the tested antioxidants, only NAC has wide clinical applications due to safety and efficacy and is available as an over-the-counter dietary supplement. In this article, we review chemoprevention studies in *Atm* deficient mice and, in more detail, our findings on the effect of NAC. The short-tem study showed that NAC suppressed genome rearrangements linked to cancer. The long-term study demonstrated that NAC reduced both the incidence and multiplicity of lymphoma.

## Introduction

Ataxia Telangiectasia (AT) is an autosomal recessive human disorder caused by mutational inactivation of the *AT mutated* (*ATM*) gene. It is a severe pleiotropic disease characterized by early onset progressive neurological degeneration, high incidence of cancer, immunodeficiency, oculocutaneous telangiectasias, growth retardation, endocrine abnormalities, infertility, and hypersensitivity to ionizing radiation (Boder, 1985; Boder et al. 1970; Gatti, 2001; Lavin et al. 1997; Meyn, 1999). The most prominent neuropathological manifestation of AT is atrophy of the cerebellar cortex associated with the loss of Purkinje and granule cells. An early sign of neurological degeneration is ataxia characterized by unstable gait and lack of coordination of head and eyes. About 40% of AT patients develop cancer, mostly in the lymphoid organs early in life and solid tumors at later age (Taylor et al. 1996; Xu, 1999). AT patients display a variety of lymphoid tumors including non-Hodgkin’s lymphoma, Hodgkin’s lymphoma and several types of leukemia, most tumors being of T cell origin. AT patients suffer from increased mortality due to malignancy, infections of the respiratory system and various rare complications (Boder, 1975; Crawford et al. 2006). It is possible to alleviate some of the clinical symptoms of AT. For example, sinopulmonary infections respond well to antibiotics or modest improvements in neurological symptoms can sometimes be achieved by L-DOPA derivatives, dopamine agonists or steroids (Lavin et al. 2007). However, currently there is no therapy available to prevent cancer or progressive neurodegeneration. The median survival of AT patients is calculated to be 19–25 years (Crawford et al. 2006).

The gene defective in AT, *ATM,* encodes a phosphatidylinositol-3′ related kinase that is involved in cell cycle checkpoint and repair responses to DNA double-strand breaks (DSBs) via a series of phosphorylated intermediary proteins including p53, Chk2, Brca1 and Nbs1 (Lavin et al. 2005; Savitsky et al. 1995; Shiloh, 2003). A lack of *ATM* function results in genomic instability characterized by chromosome breaks, chromosome gaps, translocations and aneuploidy (Cohen et al. 1975; Gropp et al. 1967; Stumm et al. 2001). *ATM* deficiency is also associated with elevated oxidative stress. *ATM* deficient cells in culture are more sensitive to oxidative stress than normal cells, cells isolated from AT patients display elevated oxidative damage to lipids and DNA and AT patients have reduced plasma antioxidant concentrations (Reichenbach et al. 2002; Reichenbach et al. 1999; Yi et al. 1990). Further evidence that AT is linked to oxidative stress stems from studies with *Atm* deficient mice. *Atm* deficient mice exhibit elevated levels of reactive oxygen species (ROS), oxidative damage to proteins and DNA, lipid peroxidation and alterations in the levels and function of antioxidative enzymes (Barlow et al. 1999; Ito et al. 2007; Kamsler et al. 2001; Quick et al. 2001; Reliene et al. 2004b). *Atm* deficient mice largely recapitulate the human disease (Barlow et al. 1996; Borghesani et al. 2000; Elson et al. 1996; Xu et al. 1996). Similar to human AT phenotype, *Atm* deficient mice display growth retardation, infertility, immunodeficiency, radiosensitivity and malignant lymphomas (Barlow et al. 1996; Elson et al. 1996; Xu et al. 1996). Although *Atm* deficient mice do not show the gross cerebellar degeneration that characterizes the human disease, more subtle alterations in the cerebellum have been observed and are consistent with a mild decrease in their motor performance (Barlow et al. 1996; Borghesani et al. 2000; Kuljis et al. 1997).

Since oxidative stress has been evidenced in AT and oxidative stress is linked to neurodegenerative diseases and cancer, it has been suggested that it may contribute to neuropathological and malignant phenotype of AT, while antioxidants might alleviate these symptoms (Barzilai et al. 2002). This hypothesis has been tested in *Atm* deficient mice in several studies that examined the effect of anti-oxidants EUK-189, tempol, CTMIO and NAC (Browne et al. 2004; Gueven et al. 2006; Ito et al. 2007; Reliene et al. 2006; Schubert et al. 2004). In addition to antioxidants, dexamethasone (Dx), which is used in chemotherapy of hematological malignancies, was studied for cancer chemoprevention in *Atm* deficient mice (Yan et al. 2002).

## Chemopreventive Agents

### NAC

NAC is a low molecular weight thiol-containing molecule that is readily taken up by the cells (Kelly, 1998). It directly inhibits reactive electrophiles and ROS and can enhance the synthesis of glutathione (GSH) as a precursor of cysteine (De Flora et al. 2001). NAC has been used in the clinical practice more than 40 years and has found wide applications (Decramer et al. 2005; Kelly, 1998; Van Schooten et al. 2002). NAC has been used for the treatment of respiratory diseases as a mucolytic agent (Webb, 1962), for acetaminophen overdose, where it rescues from GSH depletion in the liver (Prescott et al. 1977), and is available as an over-the-counter dietary supplement. NAC is most frequently taken orally and thus, we examined the effect of NAC on *Atm* deficient mice by the oral route (Reliene et al. 2004b; Reliene et al. 2006). We gave NAC supplemented drinking water to *Atm* deficient mice from fertilization throughout their life. In this treatment scenario, *Atm* ^+^/^−^ mice were crossed with each other and dams were given NAC-containing drinking water throughout pregnancy and lactation. After weaning (at about 3 weeks of age) animals continued to receive NAC in their drinking water. The major reason to start antioxidant administration as early as from fertilization was to protect against genome rearrangements that can occur during mouse development and lead to carcinogenesis later in life.

### Effect of NAC on cancer prevention

**Figure 1:**
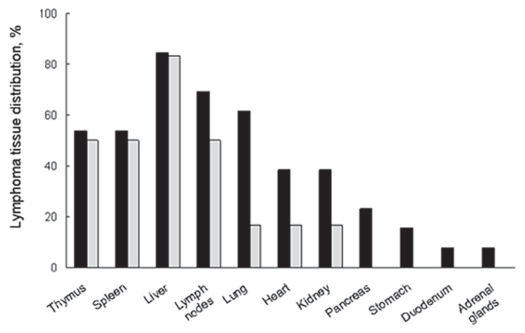
Lymphoma tissue distribution in untreated and NAC treated *Atm* deficient mice Only mice that had lymphoma are included in the calculation. Black bars depict untreated mice, gray bars show NAC treated mice. Lymph nodes affected were mesenteric and/or peripheral, thoratic and perirenal. Lymphoma in the heart was seen in epicardium and/or pericardium. Taken from (Reliene et al. 2007).

We found that NAC intake significantly increased the lifespan and reduced both the incidence and multiplicity of lymphoma in *Atm* deficient mice (Reliene et al. 2006). The mean survival of NAC treated mice was 68 weeks, while that of untreated mice was only 50 weeks (p = 0.03). We completed gross necropsy and histopathological examination to determine a possible cause of death. Consistent with previous studies, the most frequent tumor in *Atm* deficient mice was lymphoma (Barlow et al. 1996; Elson et al. 1996; Xu et al. 1996). Remarkably, the incidence of lymphoma in NAC treated mice decreased by two-fold (37.5 versus 76.5%, p = 0.02). We examined the lymphoma tissue distribution and found tumors in various organs in both NAC treated and control mice (Fig. 1). However, in NAC treated *Atm* deficient mice, the multiplicity of tumors decreased from 4.6 to 2.8 tumors per mouse (p = 0.038). Lymphoma burden was similar in the thymus, spleen and liver, while in other organs, such as lymph nodes, lung, heart, kidney, pancreas, stomach, duodenum and adrenal glands there were fewer or no tumors in the NAC treatment group (Fig. 1).

Subsequently the finding that NAC extends the reduced lifespan of *Atm* deficient mice was reproduced by another group of researchers (Ito et al. 2007). NAC was administered in drinking water and treatment was started from birth. In this study, the mean survival was approximately 20 weeks and 43 weeks in untreated *Atm* deficient mice and NAC treatment group, respectively.

## Possible Mechanism of Lymphoma Prevention by NAC

**Figure 2:**
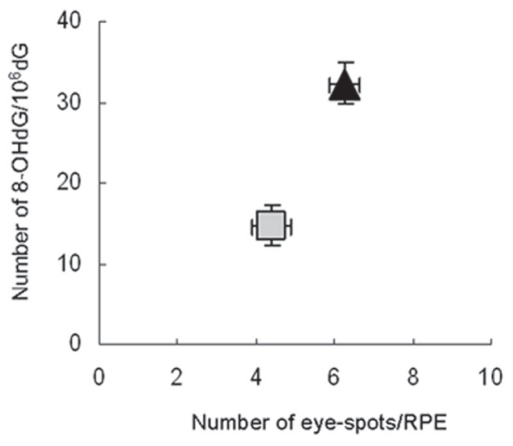
The correlation between oxidative DNA damage and the frequency of DNA deletions Oxidative DNA damage was determined as the number of oxidized guanine residues per 10^6^ guanine residues (8-OHdG/10^6^dG) using HPLC. The frequency of DNA deletions was determined as the number of eye-spots in the retinal pigment epithelium (RPE) of the eye. The eye-spots are derived from 70 kb DNA deletions at the *pink-eyed unstable* (*p*^un^) locus of the *pink-eyed dilution* (*p*) gene, which result in black pigment accumulation in the affected cells (Reliene et al. 2004a). Data for untreated mice are shown by a black triangle; results for NAC treated mice are shown by a gray rectangle. Taken from (Reliene et al. 2007).

Several studies examined the molecular action mechanism of NAC in cancer prevention in *Atm* deficient mice. NAC reduced abnormally high DNA synthesis and ROS levels in lymphocytes from *Atm* deficient mice (Ito et al. 2007; Yan et al. 2001). ROS causes oxidative DNA damage, while upregulated DNA synthesis results in a lack of time required for repair of damaged DNA template before it is used for replication. Oxidative DNA damage is often translated into irreversible genome rearrangements during replication (Kuzminov, 2001). We found that NAC suppressed both oxidative DNA damage and DNA deletions in *Atm* deficient mice supporting the interpretation that DNA deletions may be a consequence of abnormal DNA synthesis and oxidative damage (Fig. 2) (Reliene et al. 2004b). These studies showed that NAC may reduce cancer incidence by reducing oxidative stress and genomic instability.

Other studies demonstrated that NAC reduces the number of aberrant V(D)J rearrangements between T cell receptor (TCR) β and γ genes, which can cause lymphoma (Ito et al. 2007; Lista et al. 1997). In fact, tumors in *Atm* deficient mice exhibit abnormal TCR rearrangements suggesting that development of lymphoma may be driven by aberrant V(D)J recombination (Liyanage et al. 2000). NAC reduced the number of defective rearrangements and restored the decreased T cell numbers, which probably accounted for reduced lymphomagenesis (Ito et al. 2007).

ROS have also been proposed to be involved in tumor metastasis, a process that includes epithelial-mesenchymal transition, migration, invasion of the tumor cells and angiogenesis (Nishikawa et al. 2006; Wu, 2006). ROS can oxidize the critical target molecules and thereby play a role in the transcription and expression of genes implicated in tumor progression. NAC can counteract some effects of ROS in tumor progression. NAC has been reported to limit invasion of human bladder cancer cells by inhibiting both the production and activity of matrix metalloproteinase-9 involved in cancer invasion and metastasis (Kawakami et al. 2001). NAC inhibits vascular endothelial growth factor (VEGF) production and growth of angiogenesis-driven Kaposi’s sarcoma in nude mice (Albini et al. 2001), promotes anti-angiogenic factor angiostatin production and results in endothelial apoptosis and vascular collapse in an experimental breast cancer assay (Agarwal et al. 2004). We found that NAC reduced the multiplicity of lymphoma in *Atm* deficient mice, which may be explained by NAC’s anti-invasive and anti-angiogenic properties (Reliene et al. 2006). The effect was most pronounced in nonlymphoid organs supporting the observation of other studies that NAC exhibits an anti-metastatic effect.

The studies reviewed in this article show that NAC significantly reduces lymphomagenesis in *Atm* deficient mice but these positive effects by no means suggest that NAC administration can replace the missing Atm protein. It has been recently reported that ATM prevents cancer progression through detection and response to oncogene-induced DNA replication stress and DNA damage (Bartkova et al. 2005). In this report, NAC had only a marginal effect suggesting that DNA damage caused by hyperproliferative oncogenic stimuli cannot be suppressed by antioxidants. Similarly in our studies the cancer frequency and multiplicity were significantly reduced but still much elevated above wildtype levels.

### EUK-189

EUK-189, a salen-manganese compound with catalase and superoxide dismutase activities, has been previously shown to be neuroprotective in animal models characterized by oxidative damage (Doctrow et al. 2002; Melov et al. 2001). *Atm* deficient mice were treated with EUK-189 from 40 days of age via an osmotic pump implanted subcutaneously. The EUK-189 treatment improved performance on a rotarod and showed a trend towards prolonged life span (p = 0.08) (Browne et al. 2004). When the study was terminated at 5 months, 31% vehicle-treated and 56% EUK-189-treated animals were still alive (Browne et al. 2004).

### Tempol

Tempol (4-hydroxy-2,2,6,6-tetramethylpiperidine-N-oxyl) is a stable nitroxide free radical and superoxide dismutase mimetic (Damiani et al. 2000; Hahn et al. 1994; Mitchell et al. 1990). Tempol detoxifies oxygen metabolites, oxidizes redox-active trace metal ions, reduces quinone radicals and, in biological systems, is itself reduced by GSH and ascorbic acid (Branca et al. 1988; Krishna et al. 1992; Krishna et al. 1996; May et al. 2005). Tempol mixed in a mouse chow was chronically administered to *Atm* deficient mice either from fertilization or from weaning (Schubert et al. 2004). Tempol had no effect, when the treatment was started from fertilization. The intake of tempol food significantly increased the life span (mean survival 62 versus 30 weeks) in the second treatment scenario. Tempol reduced ROS levels, protein oxidation and restored mitochondrial membrane potential in thymocytes implying that chemoprevention by tempol is associated with its antioxidant activity. However, tempol treatment also reduced cell number in the thymus and decreased weight gain in *Atm* deficient mice. These effects were explained by tempol anti-proliferative activity and unknown effects, respectively. A possibility of caloric restriction was ruled out, mainly because no decrease in the intake of food containing tempol was observed.

### CTMIO

Like tempol, CTMIO belongs to a class of stable nitroxide free radicals (Damiani et al. 2000; Hahn et al. 1994; Mitchell et al. 1990). The effect of CTMIO intake through drinking water was recently examined in *Atm* deficient mice (Gueven et al. 2006). The treatment was started immediately after weaning. CTMIO prolonged the survival of *Atm* deficient mice resulting in the median survival of 54 weeks versus 16 weeks. CTMIO chemoprevention mechanism does not appear to involve apoptosis, as tumors from CTMIO treated mice did not show higher levels of apoptosis compared to tumors from untreated *Atm* deficient mice. However, it was not shown whether CTMIO induced apoptosis in mice without tumors.

### Dx

Dx is a synthetic glucocorticoid hormone used in the chemotherapy of hematological malignancies (Goss, 1992; Keating et al. 1997) for its anti-inflammatory and cytotoxic effects (Greenstein et al. 2002). In a chemoprevention study with Dx, the untreated *Atm*^−^/^−^ mice died at 2.2–4.8 month of age, while 50% of Dx treated *Atm*^−^/^−^ mice survived up to 6 months when treatment was started at 4 weeks of age, and all *Atm*^−^/^−^ mice survived for 10 months when the treatment began at 2 weeks of age, implying that Dx is more effective when given during earlier postnatal stages than later (Yan et al. 2002).

## Conclusion

The effect of NAC, EUK-189, tempol, and CTMIO was studied in *Atm* deficient mice to understand whether antioxidant therapy has a potential in the management of AT. All the described compounds had some beneficial effects, particularly, in extending the life span and reducing lymphomagenesis. Of the tested antioxidants, only NAC has a long history of safety and efficacy in the clinical settings. Therefore, NAC has a strong potential to emerge as a dietary supplement against high risk of cancer in AT and possibly other oxidative stress linked disorders. At present there is an ongoing clinical trial in pediatric AT patients, where a cocktail of antioxidants including NAC, is employed (personal communication with Dr. G. Berry, Thomas Jefferson University Medical College, also see http://www.treat-at.org). The trial is being conduced in Philadelphia, PA, and is a result of the combined efforts of the national organization to Treat-AT, Dr. Gerald Berry and his colleagues at DuPont Children’s and Children’s Hospital of Philadelphia, and SHS International Ltd (Liverpool, UK). The aim of this study is to determine whether lymphocytes from AT patients show abnormal levels of ROS and increased apoptosis and whether chronic broad antioxidant therapy retards development of lymphocyte and cerebellar dysfunction or arrest destruction of these tissues.
